# Aspirin Use on Incident Dementia and Mild Cognitive Decline: A Systematic Review and Meta-Analysis

**DOI:** 10.3389/fnagi.2020.578071

**Published:** 2021-02-04

**Authors:** Hui Li, Wan Li, Xun Zhang, Xiao-Chuan Ma, Rong-Wei Zhang

**Affiliations:** ^1^Department of Gerontology and Geriatrics, The First Affiliated Hospital of China Medical University, Shenyang, China; ^2^Department of Neurology, The Ninth People's Hospital of Shenyang, Shenyang, China; ^3^Department of Gerontology and Geriatrics, The Forth People's Hospital of Shenyang, Shenyang, China; ^4^Department of Gerontology and Geriatrics, The Third People's Hospital of Shenyang, Shenyang, China

**Keywords:** aspirin, dementia, mild cognitive decline, meta-analysis, association

## Abstract

**Background:** More people with cognitive dysfunction and dementia also fall into the category of high vascular risk, for which aspirin is one of the most frequently used drugs. However, previous studies reporting that aspirin buffers against mild cognitive decline (MCI) and dementia remain controversial. We thus conducted an updated systematic review and meta-analysis to evaluate the association of aspirin use with the risk of MCI and dementia in older adults.

**Methods:** Data sources from PubMed, Embase, Web of Science, and the Cochrane Database for randomized controlled trails (RCTs) and cohort studies (published between January 1, 2000 and April 11, 2020). Relative risks (RRs) and 95% confidence intervals (95% CIs) were used to pool data on the occurrence of dementia and MCI with random-effects models.

**Results:** Of 3,193 identified articles, 15 studies (12 cohort studies and three RCTs) were eligible and were included in our analysis, which involved a total of 100,909 participants without cognitive dysfunctions or dementia at baseline. In pooled cohort studies, aspirin use did not reduce the incidence of MCI and dementia (the pooled RR = 0.97; 95% CI = 0.85–1.11; $I_{\text{for heterogeneity}}^{2}$ = 65%) compared with non-users. However, low-dose aspirin (75–100 mg/day) was associated with a decreased likelihood of developing dementia or MCI (the pooled RR = 0.75; 95% CI = 0.63–0.9; $I_{\text{for heterogeneity}}^{2}$ = 50.5%). This association existed in studies including all-cause dementia (the pooled RR = 0.82; 95% CI = 0.71–0.96) and Alzheimer's disease (AD) (the pooled RR = 0.54; 95% CI = 0.33–0.89), but not in MCI (the pooled RR = 0.58; 95% CI = 0.31–1.08). In RCTs, low-dose aspirin use was not significantly associated with less prevalence of dementia or MCI (RR = 0.94; 95% CI = 0.84–1.05; $I_{\text{for heterogeneity}}^{2}$ = 0.0%).

**Conclusions:** In cohort studies, we found that low-dose aspirin use had a higher likelihood of reducing the incidence of dementia, which was not supported by RCTs. The evidence was insufficient to fully evaluate the effect of aspirin on cognitive function and dementia. Well-designed studies and innovative approaches are therefore needed to clarify whether the use of aspirin improves cognitive function and reduces the risk of dementia.

## Introduction

The global prevalence of dementia was 50 million people worldwide in 2015, and the number of individuals with dementia could increase to 82 million in 2030 and 152 million by 2050, which will affect ~5–8% of people ≥60 years old (WHO, 2019). Alzheimer's disease (AD) is a multi-factorial, complex, and progressive neurodegenerative disorder and the most common form of dementia in the elderly, representing 50–75% of dementia patients (Aihw, 2012). After AD, vascular dementia (VaD) is the second leading cause of dementia. Previous population-based studies identified risk factors for vascular disease as targets for preventing dementia and cognitive dysfunction (Whalley and Mowat, 2007; Sanford, 2017). It is likely to involve a multi-factorial mechanism, for example, involving increasing the levels of cerebral amyloid-β (Aβ) peptide, changing the blood–brain barrier permeability, disrupting blood cholesterol homeostasis, altering brain insulin resistance, increasing oxidative stress, causing chronic inflammation, and damaging the dopamine cholinergic nervous system (Thal et al., 2008; Harrison et al., 2014; Melo, 2019).

Aspirin 75–300 mg daily has been widely used for secondary prevention of thrombotic cerebrovascular and cardiovascular disease over the past three decades. Low-dose aspirin is generally defined as 75–100 mg/day (Rands and Orrell, 2000; Jordan et al., 2020). Its biological actions involving anti-inflammatory and anti-thrombotic properties play an important role in several diseases (Marquis-Gravel et al., 2019; Zheng and Roddick, 2019). So far, two main mechanisms have concerned the potential protective effect of aspirin use on cognitive decline and dementia in older adults. One of the mechanisms suggests that aspirin reduces the related pathology effect on AD, such as degraded levels of cerebral Aβ peptide, decreased tau phosphorylation, and improved synaptic plasticity (Ferreira et al., 2014). The other mechanism indicates that aspirin may maintain cerebral blood flow and prevent stroke in patients with ischemic cerebrovascular disease and thus protect cognitive function via inhibiting cerebral damage of vascular origin (Patrono, 2015). A recent report has investigated the effects of low-dose aspirin to enhance astrocytic lysosome biogenesis and function, which is a newly studied pathway to reduce amyloid pathology in AD (Melo, 2019).

Several observational studies reported that the use of aspirin buffers against cognitive decline or dementia when it is identified in continuous low dosage for elderly women (Kang and Grodstein, 2003; Kern et al., 2012). A systematic review and meta-analysis including longitudinal studies and randomized controlled trails (RCTs) demonstrated no benefit of aspirin in preventing cognition decline and dementia (Veronese et al., 2017). Furthermore, data from two RCTs in 2020 suggested a contrary opinion on the use of aspirin for the prevention of dementia (Matsumoto et al., 2020; Ryan et al., 2020).

In clinical practice, there is an unresolved question as to whether treatment with aspirin benefits or harms cognitive function in older people. This study therefore analyzed the currently available evidence to assess the possible effects of aspirin on the development of dementia, as well as its effects on cognitive function or impairment.

## Methods

### Search Strategy

We searched PubMed, Embase, Web of Science, and the Cochrane Database between January 1, 2000 and April 11, 2020. The completed search for PubMed was as follows: “Aspirin” (MeSH terms) OR “Acetylsalicylic Acid” (title/abstract) OR “Acid, Acetylsalicylic” (title/abstract) OR “2-(Acetyloxy)benzoic Acid” (title/abstract) AND “Dementia” (title/abstract) OR “Alzheimer's” (title/abstract) OR “Alzheimer” (title/abstract) OR “Dementia, Vascular” (MeSH terms) OR “Dementias, Vascular” (title/abstract) OR “Vascular Dementias” (title/abstract) OR “Vascular Dementia” (title/abstract) OR “Cognitive” (title/abstract) OR “Cognition” (title/abstract) OR “Memory” (title/abstract) OR “Recall” (title/abstract) OR “MMSE” (title/abstract) OR “Mini mental state examination” (title/abstract) OR “Cognition” OR “Dementia” OR “Cognition Disorders” OR “Cognitive dysfunction” OR “Alzheimer disease” OR “Mental recall” (MeSH terms). The analysis was limited to humans, but we did not involve any language restriction. The investigators (HL and WL) independently examined titles and abstracts for eligibility. In case of disagreement, the full article was retrieved and the eligibility was discussed. We also reviewed cited references of the retrieved articles to identify additional published and unpublished studies. Reviews were also retrieved. In case of disagreement, the full article was retrieved and the eligibility was discussed.

### Inclusion and Exclusion Criteria

The inclusion criteria for the study were as follows: cohort study and RCT design, population-based or community-based samples, reporting data on dementia and mild cognitive decline (MCI) diagnosed using validated criteria, and participants defined aspirin use in any dose and for any duration. Trials assessing the effect of aspirin vs. control (placebo in the RCTs or no intervention in cohort studies) were also included. Results, including odds ratios (ORs), relative risks (RRs), or hazard ratios (HRs) with corresponding 95% confidence intervals (CIs), or indirect data for the calculation of the risk estimates were noted.

The exclusion criteria for the study were as follows: participants who were diagnosed with MCI or dementia at baseline, use of active controls in the control group (non-placebo in the RCTs), studies with insufficient or overlapping data (the most recent and complete data were chosen), and the full text of relevant articles could not be obtained.

### Data Extraction and Quality Assessment

Collected data included general information (first author, publication year, study design, and region), participants' characteristics (age, sex, and sample size), dosage of aspirin, diagnostic criteria of dementia, MCI or cognitive change, follow-up period, results, and adjusted confounding factors. Two investigators independently extracted data and discussed any post-extraction discrepancies. When agreement could not be reached, a third reviewer was consulted.

Based on the extracted date, the Cochrane Risk of Bias Assessment Tool (Higgins et al., 2011) was used for RCTs that has five domains: randomization, deviation from intervention, missing data, measurement of outcome, and selective reporting. The nine-item Newcastle-Ottawa scale (Wells et al., 2000) was used for observational/non-randomized studies with domain selection, comparability, and exposure/outcome.

### Statistical Analysis

If available, pooled RRs, ORs, or HRs and 95% CIs were estimated as the ratio of the association between aspirin exposure and the risk of dementia and MCI. Because the absolute risk of dementia was low in all populations, the three measures were expected to be similar estimates of relative RRs (Greenland, 1987; Adami et al., 2008).

Meta-analysis was conducted using the Stata software, version 14.0 (Stata, College Station, TX, USA). The effect size (ES) of pooled RRs with 95% CIs was calculated to evaluate the effect of aspirin on cognitive function and dementia. Heterogeneity was assessed using the Cochran's Q statistic and quantified using the *I*^2^ tests with an *I*^2^ > 50% indicating moderate-to-high heterogeneity (Higgins et al., 2003). Publication bias was assessed by Egger's weighted regression test and Begg's rank correlation test (*P* < 0.05) (Begg and Mazumdar, 1994; Egger et al., 1997).

## Results

### Literature Search and Study Characteristics

**Figure 1:**
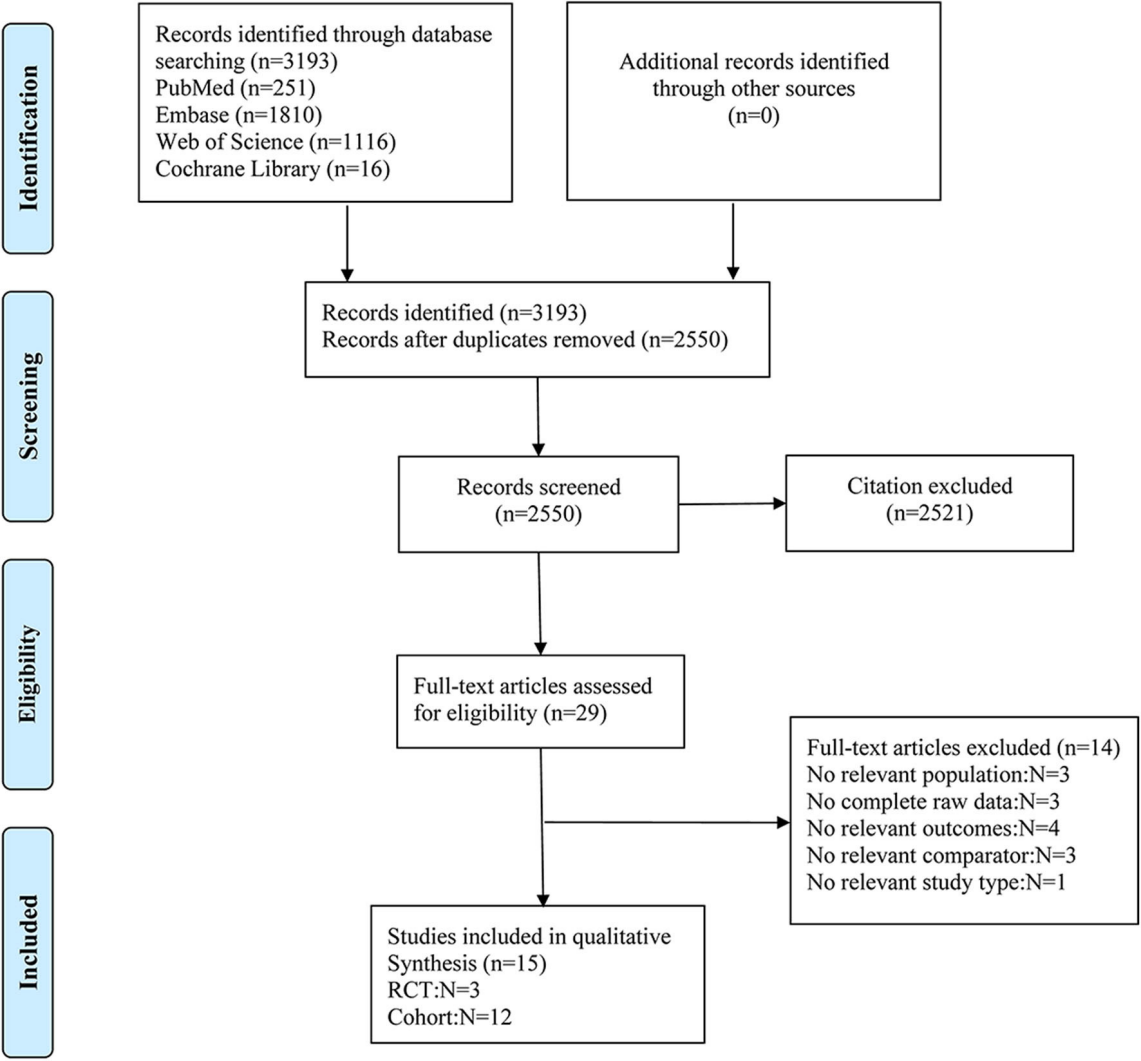
Flow chart of the evidence search and selection process.

The searches returned 3,193 articles. After excluding 643 duplicates, two reviewers separately read titles and abstracts and excluded 2,550 irrelevant citations. Twenty-nine articles were examined for full-text review, and 15 studies were included in this meta-analysis (Figure 1). Twelve included studies were cohort studies (Zandi and Breitner, 2001; Jonker et al., 2003; Kang and Grodstein, 2003; Nilsson et al., 2003; Cornelius et al., 2004; Arvanitakis et al., 2008; Szekely et al., 2008; Kern et al., 2012; Kelley et al., 2015; Chang et al., 2016; Wichmann et al., 2016; Yang et al., 2020) and three RCTs (Kang et al., 2007; Matsumoto et al., 2020; Ryan et al., 2020). Studies were conducted in the US (Zandi and Breitner, 2001; Kang and Grodstein, 2003; Kang et al., 2007; Arvanitakis et al., 2008; Szekely et al., 2008; Kelley et al., 2015; Wichmann et al., 2016), Europe (Jonker et al., 2003; Nilsson et al., 2003; Cornelius et al., 2004; Kern et al., 2012), and Asia (Chang et al., 2016; Matsumoto et al., 2020; Yang et al., 2020). However, only one RCT (Ryan et al., 2020) included both American and Australian data. Two of the studies were from Taiwan (Chang et al., 2016; Yang et al., 2020) and extracted their data from the National Health Insurance Research Database, but the investigators used different study populations and separate cohorts.

This meta-analysis included 100,909 participants (62.9% females), and the mean age was >65 years. The sample sizes varied from 612 to 23,915. The follow-up period ranged from 2 to 15 years. Most of the studies adjusted for important confounders, such as age, sex, and education (Tables 1, 2). Critical appraisal of the included studies indicated that studies in most areas were rigorous, except that some cohort studies had potentially influential differences between groups at baseline and insufficient details on loss to follow-up (Supplementary Material).

**Table 1:** Basic characteristics of cohort studies included.

**First author, year, region**	**Study design**	**BL participants (*N*)**	**Females (%)**	**Age (years)**	**Diagnostic criteria**	**Aspirin (mg/day)**	**Follow up duration (years)**	**Risk estimates**	**Adjusted variables**
Wichmann et al. (2016), USA	Prospective cohort	2,336	58.9	68.2 ± 9.3	MCI^a^	NA	10	HR	BL age, sex, education, smoking, ever heavy drinking, myocardial infarction, stroke, self-rated health, and SF-36 MCS
Szekely et al. (2008), USA	Prospective cohort	3,229	59.8	≥65	Dementia: (NINCDS-ADRDA) (ADDTC)	NA	NA	HR	Age, sex, education, race, presence of APOEε4, and baseline 3MSE
Nilsson et al. (2003), Sweden	Prospective cohort	702	58	83.9 ± 3.1	Dementia: (DSM-III-R), (NINCDS-ARDRA) (NINDS-AIREN)	75	9	RR	Age, sex, and various cardiovascular diseases
Jonker et al. (2003), The Netherlands	Prospective cohort	612	52.8	72.7 ± 6.7	MCI^b^	≤ 100	3	OR	Age, sex, education, BL MMSE score, diabetes mellitus, (rheumatoid) arthritis, and vascular diseases
Cornelius et al. (2004), Sweden	Prospective cohort	1,301	75	≥75	Dementia: (DSM-III-R)	75–500	6	RR	Age, sex, and education
Chang et al. (2016), Taiwan	Retrospective cohort	13,596	50.9	65.2 ± 8.2	Dementia: (ICD-9-CM)	<40 40–80 ≥80	8	HR	Age, sex, history of stroke, types of anti-diabetic drugs, statins, anti-hypertensive drugs, and CCI score
Arvanitakis et al. (2008), USA	Prospective cohort	1,019	69.4	75 ± 7.2	Dementia: (NINCDS-ADRDA)	NA	12	HR	Age, sex, and education
Yang et al. (2020), Taiwan	Retrospective cohort	6,028	57.4	73.1 ± 5.7	Dementia (ICD-9-CM)	75	5.98 ± 3.5	HR	Age, sex, COPD, DM, HTN, IHD, CHF, CVA, and CRI
Kern et al. (2012), Sweden	Prospective cohort	789	100	≥70	Dementia (DSM-III-R)	75–160	5	RR	NA
Zandi and Breitner (2001), USA	Prospective cohort	3,227	58.2	73.9 ± 6.4	Dementia (DSM-III-R)	NA	≥2	HR	Age, sex, education, and APOE gene
Kelley et al. (2015), USA	Prospective cohort	23,915	43	64 ± 9.2	MCI^c^	NA	5.9	OR	Age, sex, race, education, income, and region
Kang and Grodstein (2003), USA	Prospective cohort	16,128	100	≥70	MCI^d^	NA	15	RR	Age, education, years between first and second interview, BMI, diabetes, postmenopausal hormone use, age at menopause, anti-depressant use, vitamin E use, other NSAID use, hypertension, mental health index, smoking, energy–fatigue index from medical outcomes short form 36, current alcohol, and heart disease

*MCI, mild cognitive impairment; BL baseline; DSM-III-R, Diagnostic and Statistical Manual of Mental Disorders, revised 3rd edition; NINCDS-ADRDA, National Institute of Neurological and Communicative Disorders and Stroke and the Alzheimer's Disease and Related Disorders Association; NINDS-AIREN, National Institute of Neurological Disorders and Stroke-Association International pour la Recherce et l'Enseigmnent en Neurosciences; ICD-9-CM, International Classification of Diseases, Revision Ninth, Clinical Modification; ADDTC, the State of California Alzheimer's Disease Diagnostic and Treatment Centers criteria; NA, not available; RR, relative risk; OR, odds ratio; HR, hazard ratio; BMI, body mass index; CCI, Charlson–Deyo comorbidity index; COPD, chronic obstructive pulmonary disease; 3MSE, Modified Mini-Mental State; NSAID, non-steroidal anti-inflammatory drug; SF-36 MCS, Short Form Health Survey Mental Component Score; MMSE, Mini-Mental State Examination; CHF, congestive heart failure; CRI, chronic renal insufficiency; CVA, cerebrovascular accident; DM, diabetes mellitus; HTN, hypertension; IHD, ischemic heart disease. MCI^a^ (Self- or proxy-reported diagnosis); MCI^b^ (Edwards–Nunnally method for three cognitive tests); MCI^c^ (six-item screener <5); MCI^d^ (six items of cognitive test)*.

**Table 2:** Basic characteristics of the randomized controlled trials included.

**First author, year, region**	**BL participants (*N*)**	**Females (%)**	**Age (years)**	**Diagnostic criteria**	**Aspirin (mg/day)**	**Follow-up duration (years)**	**Risk estimates**	**Adjusted variables**
Matsumoto et al. (2020), Japan	2,536	45.3	65 ± 10	Dementia: (1) Prescription of anti-dementia drugs (2) Admission to hospital/nursing home due to dementia	81–100	Median 11.4	HR	Age, sex, hypertension, dyslipidemia, smoking, HbA1c, and BMI
Kang et al. (2007), USA	6,377	100	66.2 ± 4.1	Tests for cognitive function: global score, verbal memory score, TICS test, and category fluency MCI: worst 10% of distribution of decline from baseline (global score −0.8 points, verbal memory score −0.9 points, TICS −4 points, category fluency −7 points)	100 (alternate day)	Mean 9.6 (range 8.2–11.3)	RR	NA
Ryan et al. (2020), USA and Australia	19,114	56.4	Median 74	Dementia: DSM-IV-R NIA-AA	100	Median 4.7 (range 3.6–5.7)	HR	NA

*MCI, mild cognitive impairment [worst 10% of distribution of decline from baseline (global score −0.8 points, verbal memory score −0.9 points, TICS −4 points, category fluency −7 points); BL, baseline; TICS, Telephone Interview for Cognitive Status; DSM-IV, Diagnostic and Statistical Manual of Mental Disorders, revised 4th edition; NIA-AA, National Institute on Aging—Alzheimer's Association; NA, not available; RR, relative risk; HR, hazard ratio; BMI, body mass index. Cognitive function (global score, verbal memory score, TICS test, category fluency)*.

### Aspirin Use and Incident Dementia or MCI

#### Cohort Studies

**Figure 2:**
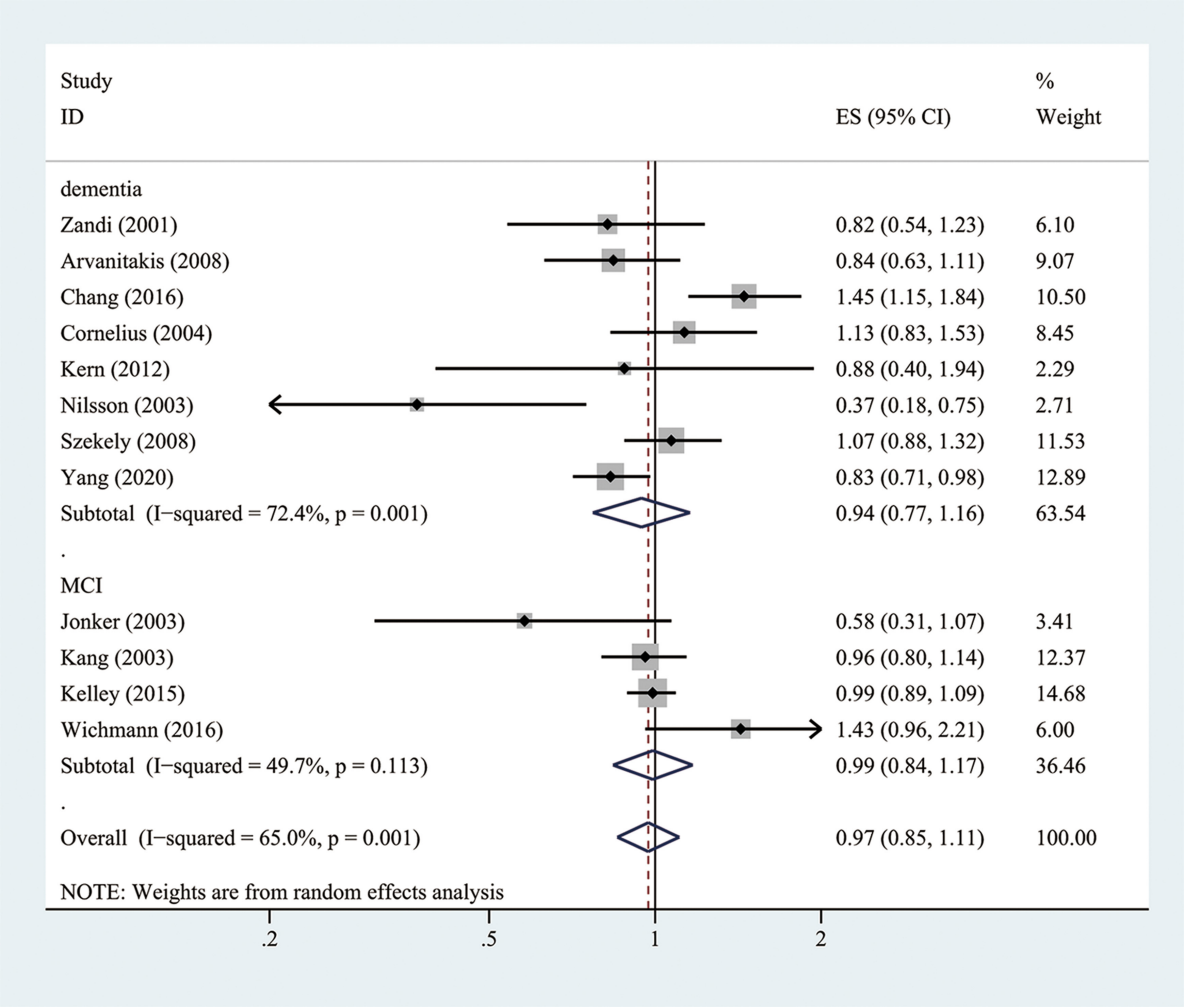
Association of aspirin use with incident dementia or mild cognitive decline in cohort studies.

Twelve cohort studies (10 prospective studies and two retrospective studies) investigated the association between aspirin use and the risk of dementia or MCI (Zandi and Breitner, 2001; Jonker et al., 2003; Kang and Grodstein, 2003; Nilsson et al., 2003; Cornelius et al., 2004; Arvanitakis et al., 2008; Szekely et al., 2008; Kern et al., 2012; Kelley et al., 2015; Chang et al., 2016; Wichmann et al., 2016; Yang et al., 2020). Overall, when adjusted for confounders, aspirin use showed no significant decreased risk of developing dementia or MCI (the pooled RR = 0.97; 95% CI = 0.85–1.11; $I_{\text{for heterogeneity}}^{2}$ = 65%) (Figure 2). There were methodological heterogeneities due to differences in the study type (prospective vs. retrospective studies), follow-up duration, and dosage.

**Figure 3:**
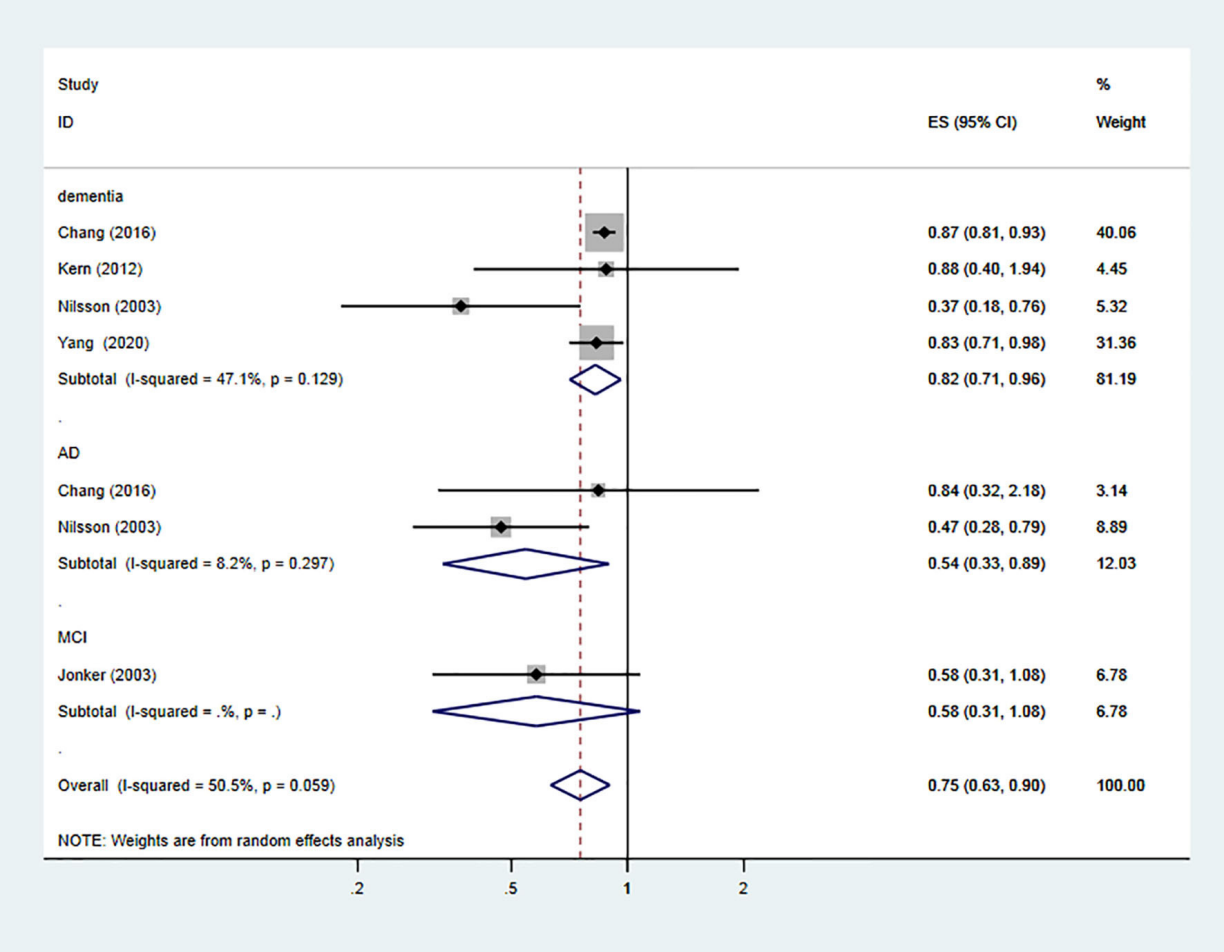
Association of low-dose aspirin use with incident dementia or mild cognitive decline in cohort studies.

However, after adjusting for low-dose aspirin, the results showed a protective effect against the risk of dementia and MCI (the pooled RR = 0.75; 95% CI = 0.63–0.9; $I_{\text{for heterogeneity}}^{2}$ = 50.5%) (Figure 3). For subgroup analysis stratified by the subtype of cognitive impairment, the results indicated that low-dose aspirin use had a protective effect against all-cause dementia (the pooled RR = 0.82; 95% CI = 0.71–0.96) and AD (the pooled RR = 0.54; 95% CI = 0.33–0.89), but not in MCI (the pooled RR = 0.58; 95% CI = 0.31–1.08) (Figure 3).

#### RCTs

**Figure 4:**
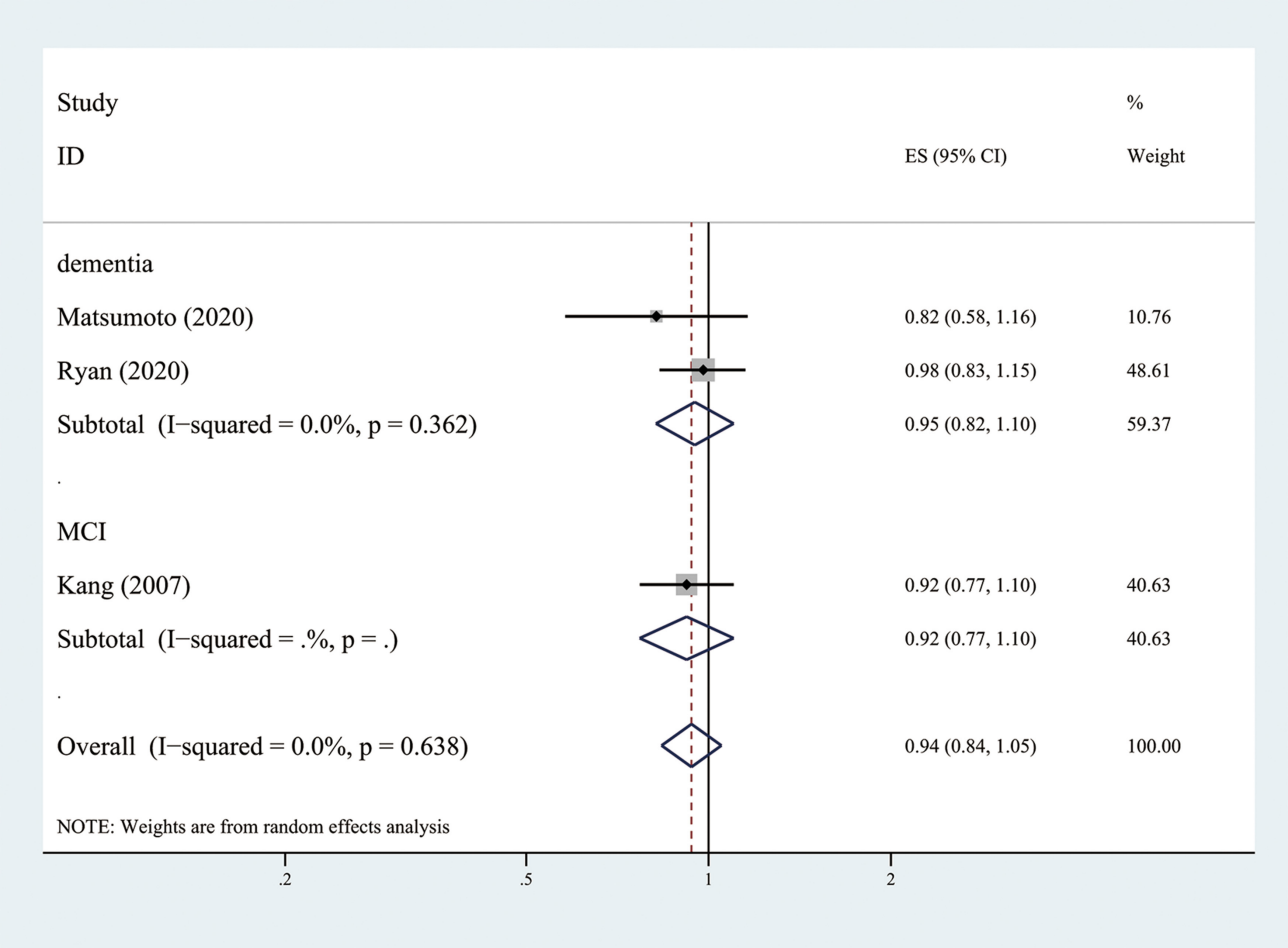
Association of low-dose aspirin use with incident dementia or mild cognitive decline in randomized controlled trails.

The three RCT (Kang et al., 2007; Matsumoto et al., 2020; Ryan et al., 2020) showed that low-dose aspirin use did not have a positive effect on reducing MCI or dementia (the pooled RR = 0.94; 95% CI = 0.84–1.05; $I_{\text{for heterogeneity}}^{2}$ = 0.0%) (Figure 4). However, the Japanese Primary Prevention of Atherosclerosis with Aspirin for Diabetes (JPAD) trial (Matsumoto et al., 2020) showed that use of low-dose aspirin resulted in a 42% lower risk of dementia compared to the placebo group in females with diabetes (*P* = 0.03).

#### Publication Bias

**Figure 5:**
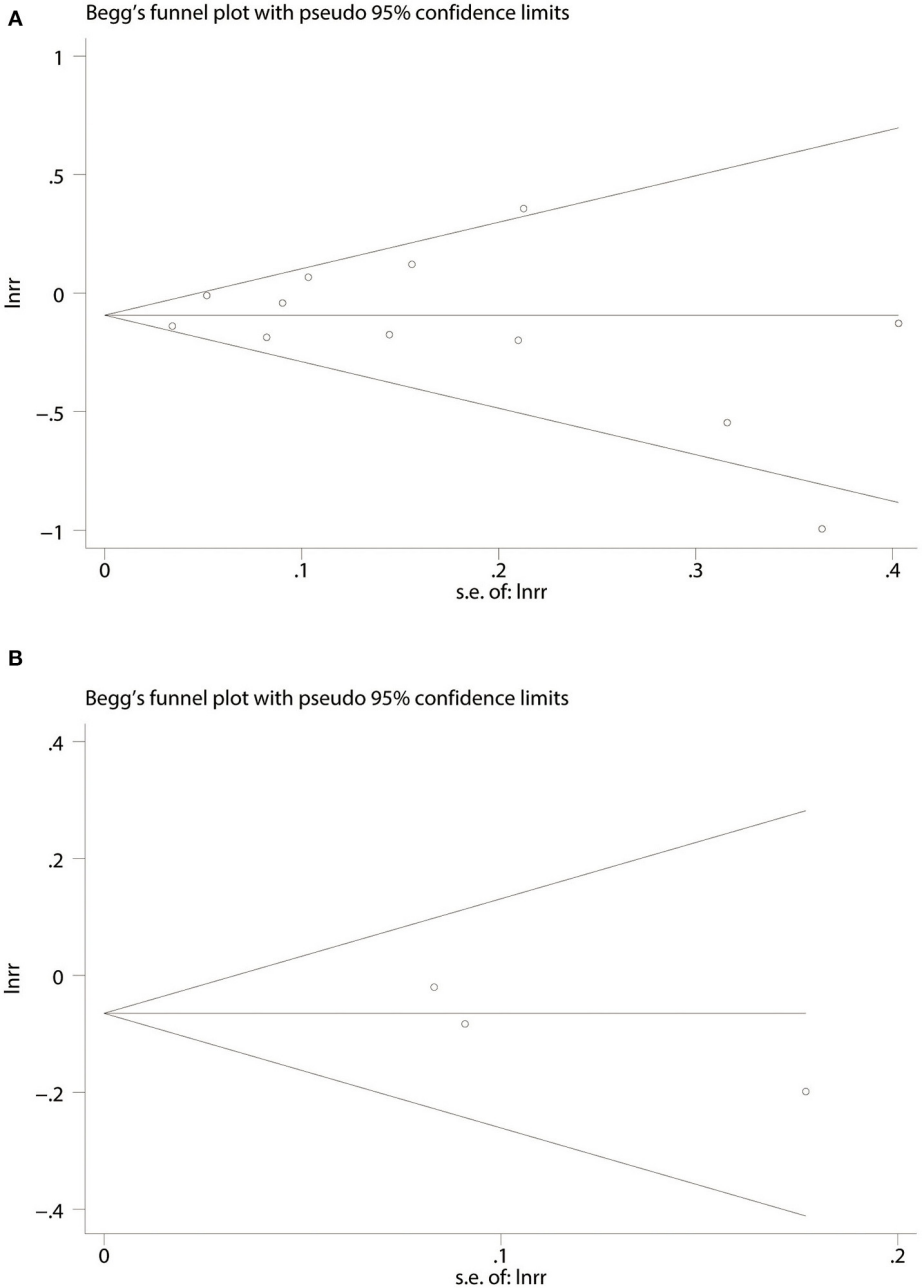
Begg's funnel plot for association of aspirin use with incident dementia or mild cognitive decline in twelve cohort studies **(A)**. Three randomized controlled trails **(B)**. RR, relative risk; lnrr, the Napierian logarithm of relative risk; s.e. of lnrr, standard error of lnrr.

Twelve cohort studies (*P* = 0.837 and *P* = 0.923) and three RCTs (*P* = 0.296 and *P* = 0.280) according to Begg's and Egger's tests, respectively (Figures 5A,B), suggested no evidence of publication bias in all publications.

## Discussion

Our results of 15 pooled studies indicated that the use of any dose of aspirin was not associated with a lower risk of developing dementia or MCI. When restricted to adjusting the dosage for low-dose aspirin, it was associated with a 25% (95% CI = 10–37) reduction in the risk of incident dementia and MCI in five cohort studies. Nevertheless, across RCTs, the results did not support the use of low-dose aspirin to improve general cognitive test results or dementia incidence among the elderly population.

In cohort studies, discrepancies regarding the effects of different aspirin dosages suggested that low-dose aspirin might have a protective effect against dementia and cognitive decline through two main mechanisms: (i) effect on AD-related pathology and (ii) protection against vascular accidents that may subsequently generate VaD or vascular cognitive impairment (VCI).

Aspirin reduces the related pathology effect on AD. First, in animal models, because platelets are the main source of systemic Aβ and amyloid precursor protein (APP), low-dose aspirin might directly reduce the amount of circulating Aβ derived from platelets (Bush et al., 1990; Inyushin et al., 2019). Chandra et al. (2018) reported a new function of low-dose aspirin in mediating peroxisome proliferator-activated receptor alpha (PPARα), which upregulated transcription factor EB, leading to increased astrocytic lysosome biogenesis and functioning in the brain, leading to enhanced clearance of Aβ. Second, Khezrian et al. (2020) and Sato et al. (2004) reported that low-dose aspirin use might slow the progression or occurrence of white matter lesions (WMLs) and might help to preserve cognition in the elderly population. WMLs are common findings in AD (Richard et al., 2010), and Ferrari et al. (2018) reported that low-dose aspirin therapy slowed AD progression, especially among non-apolipoprotein E allele carriers via probable modulation of the WML burden on AD patients.

Aspirin has a protective effect against vascular accidents that may lead to VaD or vascular cognitive decline. First, experimental data showed that low-dose aspirin mainly resulted in an anti-platelet effect, which might play an important role in the protection of cognitive function by enhancing the cerebral blood flow in the cognitive area, to prevent transient cerebral ischemic attacks and maintain blood–brain barrier integrity (Ridker et al., 1996; Patrono, 2015). In addition, the limited anti-inflammatory action of low-dose aspirin may help reduce neurodegenerative diseases, which does not necessarily require inhibition of COX-2 in inflammatory cells, which occurs with high daily doses of aspirin, but it may simply reflect its anti-platelet effect (Patrignani and Patrono, 2015). Furthermore, aspirin-triggered lipoxins reduce the impact of neuroinflammation on the development of cerebral small vessel disease (cSVD) by mitigating the neurotoxicity and stimulating alternative activation of microglia (Kaiser et al., 2014; Patrono, 2015). Finally, translational models indicated aspirin may maintain cerebral blood flow and prevent stroke in patients with ischemic cerebrovascular disease, thereby protecting against VCI and VaD by inhibiting vasogenic brain damage (Hainsworth et al., 2017).

Importantly, previous studies have shown that the pathology of typical dementia occurs 20 years before the clinical onset (Reiman et al., 2012), so low-dose aspirin may produce a sustained protective effect in participants only if they have taken aspirin for a sufficient duration, which probably explains the negative effect of pooled RCTs on the prevention of dementia and cognitive dysfunction. One of the RCTs, the ASPREE trial (Ryan et al., 2020), found that low-dose aspirin use showed no significant decrease in the risk of developing dementia, with a median follow-up period of 4.7 years, just 5 months ahead of schedule. However, the JPAD trial reported a positive effect in females with a median follow-up of 11.4 years, and Matsumoto et al. (2020) reported that the cumulative incidence of dementia increased significantly during a follow-up of 8 years. These findings contribute to the existing literature that has minimized biases about low-dose aspirin therapy because of insufficient treatment durations, facilitating stronger conclusions about the specificity of low-dose aspirin for incident dementia and cognitive changes.

Furthermore, some important confounding and potential risk factors should be considered. For example, previous studies have shown that poor socioeconomic status (Prince et al., 2012), social relationships (Kuiper et al., 2015), and behavioral lifestyle (Kivipelto et al., 2018) might affect the development of dementia and cognitive decline. Recently, the Harvard Aging Brain Study (Biddle et al., 2020) suggested that widowhood was an underrecognized risk factor associated with AD-related cognitive decline and impairment. In addition, a large nationwide case-control study (Savolainen-Peltonen et al., 2019) and RCT (Shumaker et al., 2004) indicated that postmenopausal hormone therapy could be a potential risk factor for cognitive change in elderly females. The actual effects of aspirin might therefore be understated or overstated by these potential confounding or underrecognized risk factors.

To the best of our knowledge, this meta-analysis is the most comprehensive one in the field of evidence-based medicine (EMB). In 2000, Rands and Orrell (2000) failed to select the eligible RCTs for an intervention review between aspirin and VaD. In 2015, Wang et al. (2015) conducted a meta-analysis of eight cohort studies and three case-control studies characterizing the association of aspirin use and AD risk, which concluded that there was a significant reduction in the risk of AD. In 2017, Veronese et al. (2017) summarized the data from five cohort studies and three RCTs, to show no protective effect of low-dose aspirin on cognitive function; however, only limited studies were involved. Compared to previous studies, our updated meta-analysis did not include case-control studies because they were less adept at showing a causal relationship than cohort studies or RCTs, and case-control studies were more prone to bias, especially when reporting bias and recall bias. Furthermore, the meta-analysis provides more comprehensive evidence, including a relatively larger number of studies (*n* = 15), which were not included in previous studies. Additionally, according to the Cochrane handbook, we conducted a rigorous and extensive literature search for all eligible studies in large databases, including PubMed, Embase, Web of Science, and the Cochrane Database. Finally, we did not use any language restriction in retrieval processing, so it is unlikely that we missed important articles.

However, there are several potential limitations, which have to be addressed in interpreting the results of our meta-analysis. First, the possibility of selection bias in observational studies included the fact that a percentage of participants that replaced aspirin with non-aspirin non-steroidal anti-inflammatory drugs (NSAIDs), or other active drugs, might have created another important bias. Second, in cohort studies, although we conducted a subgroup analysis stratified by the subtypes of dementia (all-cause dementia and AD), it failed to analyze VaD, so the limited number of studies of each outcome and the effects of aspirin might be different depending on the subtype. Third, variability in diagnostic criteria of dementia and cognitive decline between data sets may have affected our results. Finally, although the important potential confounders were adjusted in the pooled analyses, statistical adjustment for the studies may not entirely resolve these problems. For example, the indications for aspirin may be different across countries, other comorbidities, and other medications, including anti-hyperglycemic drugs, antidepressants, and non-aspirin NSAIDs, which may influence the conclusions.

## Conclusions

Our meta-analysis of observational studies and a single RCT indicated that low-dose aspirin use over long periods might protect against the occurrence of dementia and MCI. However, all pooled RCTs did not support a preventive effect. Taking into account the controversial results between observational studies and RCTs, well-designed studies and innovative approaches are required in future long-term studies to determine whether low-dose aspirin has the ability to decrease the prevalence of MCI and dementia.

## Data Availability Statement

The original contributions presented in the study are included in the article/Supplementary Materials, further inquiries can be directed to the corresponding author/s.

## Author Contributions

HL drafted the rough manuscript. R-WZ and WL designed the study. Literature search and selection were conducted by XZ and X-CM. All authors have read and approved the final manuscript.

## Conflict of Interest

The authors declare that the research was conducted in the absence of any commercial or financial relationships that could be construed as a potential conflict of interest.
