# Supplementary figures and images for: Aspirin Use on Incident Dementia and Mild Cognitive Decline: A Systematic Review and Meta-Analysis
Source: Front Aging Neurosci. 2021 Feb 4;12:578071. doi: 10.3389/fnagi.2020.578071 (PMC7890199; doi:10.3389/fnagi.2020.578071)

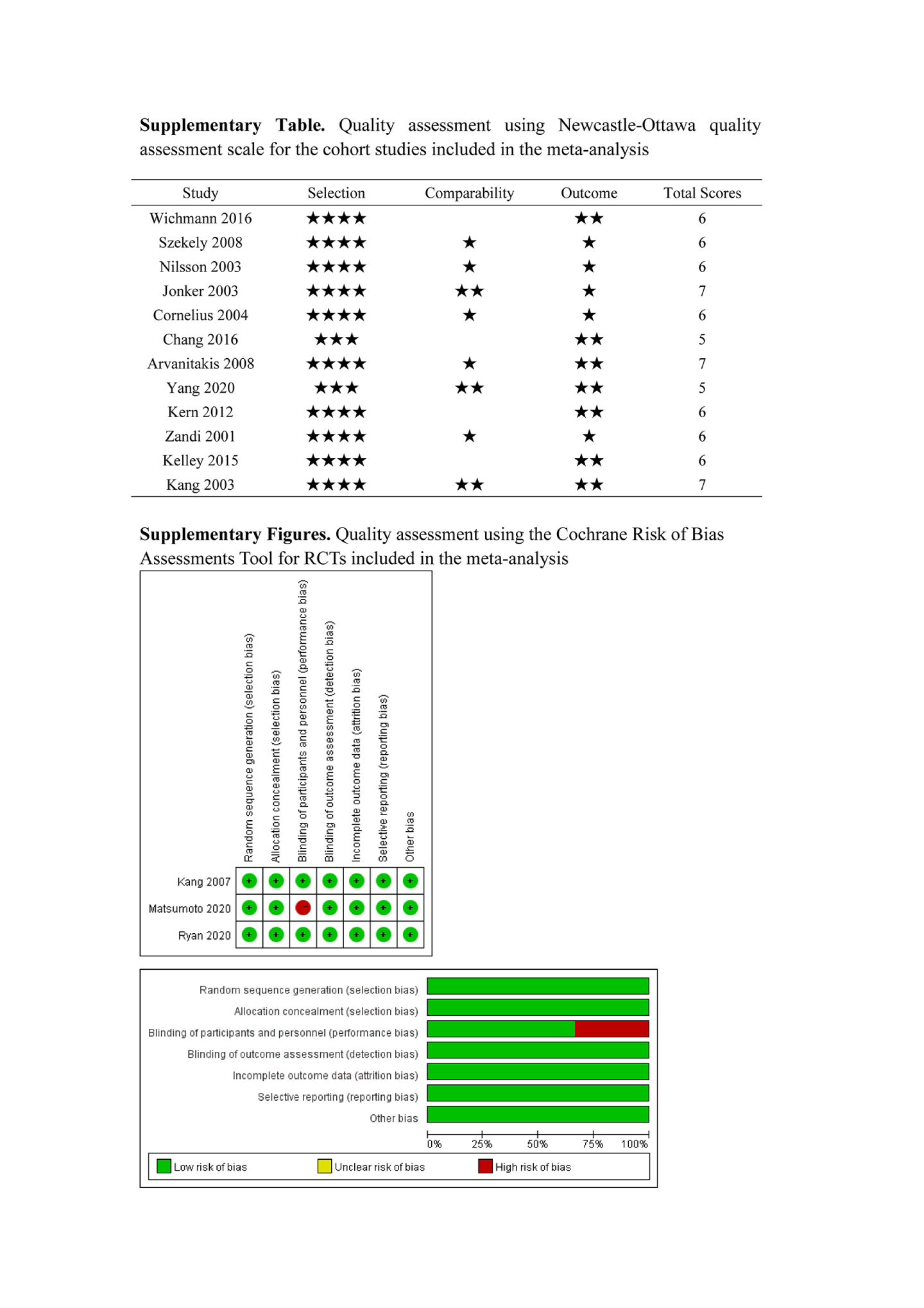

Supplement: Supplementary file 1 [file Image_1.tif]
